# Portal Vein Thrombosis in Liver Transplantation: A Retrospective Cohort Study

**DOI:** 10.3390/jcm12123951

**Published:** 2023-06-09

**Authors:** Luis Manuel Barrera-Lozano, Jaime Alberto Ramírez-Arbeláez, Cristian Leonardo Muñoz, Jorge Andrés Becerra, Luis Guillermo Toro, Carlos M. Ardila

**Affiliations:** 1 Transplant Department, Hospital San Vicente Fundación, Rionegro 054047, Colombia; manuel.barrera@udea.edu.co (L.M.B.-L.); jaime.perez@sanvicentefundacion.com (J.A.R.-A.); cristian.munoz@sanvicentefundacion.com (C.L.M.); luis.toro@sanvicentefundacion.com (L.G.T.); 2 Vascular Medicine Department, Faculty of Medicine, Universidad de Antioquia UdeA, Medellín 050010, Colombia; 3 Foscal Clinic, Floridablanca 681003, Colombia; andresbecerraromero@gmail.com; 4 Basic Studies Department, School of Dentistry, Universidad de Antioquia UdeA, Medellín 050010, Colombia

**Keywords:** liver transplantation, liver cirrhosis, portal vein thrombosis, survival

## Abstract

Portal vein thrombosis was considered a contraindication for liver transplantation. This study analyzes the perioperative complications and survival of liver transplant patients with portal vein thrombosis (PVT). A retrospective observational cohort study of liver transplant patients was conducted. The outcomes were early mortality (30 days) and patient survival. A total of 201 liver transplant patients were identified and 34 (17%) patients with PVT were found. The most frequent extension of thrombosis was Yerdel 1 (58.8%), and a portosystemic shunt was identified in 23 (68%) patients. Eleven patients (33%) presented any early vascular complication, PVT being the most frequent (12%). The multivariate regression analysis showed a statistically significant association between PVT and early complications (OR = 3.3, 95% confidence interval 1.4–7.7; *p* = 0.006). Moreover, early mortality was observed in eight patients (24%), of which two (5.9%) presented Yerdel 2. For Yerdel 1, patient survival according to the extent of thrombosis was 75% at 1 year and 3 years, while for Yerdel 2, it was 65% at 1 year, and 50% at 3 years (*p* = 0.04). Portal vein thrombosis significantly influenced early vascular complications. Furthermore, portal vein thrombosis Yerdel 2 or higher impacts the survival of liver grafts in the short and long term.

## 1. Introduction

Portal vein thrombosis refers to the formation of a blood clot within the portal vein, which is the major vein responsible for carrying blood from the gastrointestinal tract, spleen, and pancreas to the liver [1,2,3]. This condition disrupts normal blood flow and can lead to complications. The risk factors for portal vein thrombosis can be classified into local and systemic factors [1,2,4,5]. The local risk factors are: (1) Liver cirrhosis: The most common cause of portal vein thrombosis is where scarring of the liver disrupts blood flow and promotes clot formation. (2) Liver cancer: Tumors in the liver can compress or invade the portal vein, increasing the risk of clot formation. (3) Inflammatory bowel disease: Conditions such as Crohn’s disease or ulcerative colitis can cause inflammation and damage to the blood vessels, increasing the likelihood of clot formation. (4). Abdominal or liver surgery: Surgical procedures in the abdominal area can damage the portal vein or alter blood flow, predisposing to clot formation. (5). Pancreatitis: Inflammation of the pancreas can lead to local clot formation, affecting the nearby portal vein. Instead, the systemic risk factors include [1,3,5]: (1) Inherited or acquired prothrombotic disorders: Certain genetic or acquired conditions, such as Factor V Leiden mutation, protein C or S deficiency, antiphospholipid syndrome, and myeloproliferative neoplasms, increase the risk of blood clot formation throughout the body, including the portal vein. (2) Hypercoagulable states: Conditions that cause an abnormal increase in blood clotting factors, such as pregnancy, oral contraceptive use, hormone replacement therapy, or prolonged immobility, can predispose individuals to portal vein thrombosis. (3) Infection: Certain infections, including liver abscesses or intra-abdominal infections, can trigger clot formation in the portal vein. (4) Dehydration: Reduced blood flow and increased blood viscosity due to dehydration can contribute to clot formation. (5) Trauma: Severe abdominal or liver trauma can damage the portal vein and initiate clotting. There have even been reports of portal vein thrombosis related to abdominal trauma [6] and injuries associated with sports activities [7].

It is important to note that in some cases, the cause of portal vein thrombosis may remain unknown (idiopathic). Therefore, prompt diagnosis and appropriate management of portal vein thrombosis are crucial to prevent complications such as liver damage, portal hypertension, and bowel ischemia [2,3,4,5].

The time to thrombus formation (acute or chronic) classifies portal vein thrombosis. Acute portal vein thrombosis can cause abdominal pain, intestinal ischemia/infarction, and even death; however, chronic portal vein thrombosis can be clinically asymptomatic [8]. It is recommended that all acute cases of portal vein thrombosis be treated with anticoagulation for at least 3 months, though 6–12 months is more common in practice; however, long-term anticoagulation therapy is recommended for chronic portal vein thrombosis patients who have a permanent risk factor for thrombosis [8,9]. Furthermore, in situations of recent portal vein thrombosis, pharmaceutical thrombolysis (local or systemic) aiming at recanalization has been advocated as an adjuvant to anticoagulation. In patients with chronic portal vein thrombosis, portal vein recanalization (followed by transjugular intrahepatic portosystemic shunt) has been researched mostly in liver transplant candidates to allow a physiological anastomosis between the graft and recipient portal veins [9].

The treatment of portal vein thrombosis aims to achieve two main goals: prevent the clot from growing larger and causing further complications; and restore or maintain normal blood flow in the portal vein [1,3]. The specific treatment approach depends on various factors such as the extent and severity of the clot, the presence of underlying conditions, and the individual patient’s overall health [2,4]. Here are some common treatment options for portal vein thrombosis: anticoagulation therapy, thrombolysis, transjugular intrahepatic portosystemic shunt, surgical intervention, treatment of underlying conditions, and symptomatic management. This may include the treatment of portal hypertension-related complications such as variceal bleeding or ascites [3,4].

Portal vein thrombosis can also be a significant consideration in the context of liver transplantation. The presence of portal vein thrombosis may affect the indications and management of liver transplantation. Some indications for liver transplantation in the presence of portal vein thrombosis include decompensated liver cirrhosis with portal vein thrombosis, non-cirrhotic portal hypertension with portal vein thrombosis, and liver cancer (hepatocellular carcinoma) with portal vein thrombosis [1,2,3,4]. However, suppurative portal vein thrombosis (pylephlebitis), a rare disorder associated with an intra-abdominal infection or inflammatory process, is a contraindication to liver transplantation [10]. There have also been reports of severe pylephlebitis in patients with acute sigmoid diverticulitis [11], which is one of the most common intraabdominal infections associated with the development of portal vein thrombosis.

In the past, portal vein thrombosis was often considered a relative contraindication for liver transplantation due to the associated surgical complexity and perceived risk of poor outcomes; however, with advancements in surgical techniques and perioperative management, the perspective on portal vein thrombosis as a contraindication has evolved [12]. The first successful transplant in a patient with portal vein thrombosis was performed in 1985 [13]. Its incidence is between 2 and 26% [14,15]. Despite the advances in surgical techniques, the associated perioperative mortality is 13%, rising to 28% when the thrombus extends distally towards the superior mesenteric vein, and with one-year mortality even reaching up to 42% in cases of complete unresolved portal vein thrombosis at the time of implantation [16]. Advancements in surgical techniques, including thrombectomy, patch venoplasty, and jump grafts, have made it possible to address portal vein thrombosis effectively during transplantation. Moreover, improved perioperative care, including anticoagulation protocols and careful post-transplant monitoring, has helped mitigate the risks associated with portal vein thrombosis.

With these advancements, the presence of portal vein thrombosis is no longer an absolute contraindication for liver transplantation. Instead, the decision to proceed with transplantation in the presence of portal vein thrombosis is based on individual patient factors, including the extent and location of the thrombus, the underlying liver disease, tumor burden (if applicable), and the overall health of the patient. Each case is carefully evaluated, and a multidisciplinary team assesses the risks and benefits to determine the suitability for transplantation.

Multiple types of thrombosis classification have been used [17]; however, the classification proposed by Yerdel et al. is the most widely used [18]. Grade 1 thrombosis has similar graft survival to recipients who do not have it, compared to the most extensive thrombosis (grade 2–4), with a 50% survival rate after one year of implantation. There are multiple alternatives for its reconstruction, including thrombectomy with portal anastomosis or to the splenomesenteric confluence, eversion thromboembolectomy [19,20,21,22,23], portomesenteric bridge [12], renoportal anastomosis [23,24,25], portocaval hemitransposition [26,27], and multivisceral transplantation [17,26,28]. It is worth noting that there are other alternatives to increase portal flow, such as left gastric vein venovenous transposition [29] or portal vein arterialization [30]. These alternatives are used according to the time of diagnosis (intraoperative finding or not), the extension of the thrombus in the splenic portomesenteric system, the flow velocity of the portal vein, and the presence or absence of physiological shunts of the portal system [17].

Despite the advances mentioned above, the study hypothesis is that the portal vein thrombosis impacts the survival and early complications of patients who have undergone liver transplants. Therefore, the aim of this study was to analyze the perioperative complications and survival of liver transplant patients with portal vein thrombosis.

## 2. Materials and Methods

A retrospective observational cohort study was conducted on liver transplant patients at the San Vicente Fundación Hospital in Rionegro, Colombia, between January 2013 and April 2021. All liver transplant patients older than 14 years were included, and those with the presence of portal vein thrombosis were identified in the pre-transplant protocol or as an intraoperative finding at the time of implantation. Patients with combined liver-kidney transplants and/or multi-visceral transplants were excluded. Patients with tumor thrombi associated with hepatocarcinoma were excluded, in addition to those in whom it was not possible to classify the extent of the thrombus in the splenic portomesenteric axis, whether by images or by the findings of the surgeon’s description.

All patients who were candidates for liver transplantation underwent portal circulation duplex and triphasic computed tomography (CT) of the liver per protocol. Given a lesion suspected of hepatocarcinoma, an abdominal resonance with gadolinium (AR) was performed to characterize and confirm the presence or absence of the tumor. In cases of tumor confirmation, a new CT or AR was performed every 3 months to assess the progression of the lesion, while the patient was on the waiting list. Once the presence of thrombosis was identified, it was graded with the Yerdel et al. classification [18]. Moreover, the presence of systemic port shunts, their location, and whether they were physiological were established. The presence of a physiological shunt was defined when the diameter was greater than or equal to 1 cm [31]. In those patients in whom extensive portal vein thrombosis (Yerdel 2–4) was identified acutely during the protocol, or while on the waiting list, low molecular weight heparins were started in a prophylactic dose.

The options for the reperfusion and reconstruction of the portal vein at the time of transplantation were made at the discretion of the surgeon, and their choice depended on the extension of the thrombus, and the patency of the veins of the splenic portomesenteric axis. The suprahepatic vein implantation technique used was piggyback, and no venovenous bypass was used. The evaluation of the arterial and venous flow was carried out by the duplex of the graft, both in the immediate pop (first 6 h) and at 24 h of control, and then as necessary according to the findings in the first duplexes and the enzymatic curve. The management of the portal vein rethrombosis was performed with anticoagulation, interventional radiology, or surgery. Candidates for retransplantation were those patients with hepatic artery thrombosis in the first 7 days, or with no primary function of the graft.

The variables evaluated were classified as demographic, pre-transplant clinical, surgical, and perioperative complications. The outcomes to be evaluated were early mortality (30 days) and patient survival. Early complications include hepatic artery thrombosis, hepatic artery stenosis, portal vein thrombosis, and death within the first month. Infections were also considered within the postoperative complications. The data were taken from the database of the institutional clinical history.

This study was approved by the Institutional Bioethics Committee of the Hospital San Vicente Fundación, Rionegro, Colombia (Registration 15-2021). All the recommendations of the Declaration of Helsinki were followed.

### Statistical Analysis

The analysis included a descriptive and an analytical phase. In the descriptive phase, the qualitative and quantitative variables were studied, and their frequencies, averages, standard deviations, medians, and ranges were presented. In the analytical phase, patient survival was evaluated using the Kaplan–Meier method, and graft survival in patients with and without portal vein thrombosis was compared using the log-rank test. Some factors associated with the mortality of transplant recipients and with early complications were further explored in a bivariate analysis, adjusting the outcome with the degree of extension of portal vein thrombosis. A multivariate logistic regression analysis was also performed to assess the relationship between portal vein thrombosis and early complications. Statistical analysis was performed using the “R” program (R-4.2.2 for Windows) [32].

## 3. Results

### 3.1. Demographic and Clinical Characteristics

Table 1 presents the main demographic and clinical characteristics of the patients studied. A total of 201 liver transplant patients were collected during the study period. Thirty-four (17%) patients with portal vein thrombosis were identified, either at the time of listing (31 patients) or at the time of explant (3 patients). Fourteen (41%) patients had hepatocarcinoma; however, the thromboses were not associated with the tumor.

Of the patients with portal vein thrombosis on the waiting list, only 24% (eight patients) received anticoagulation before or during their stay on the list. Preoperative imaging or intraoperative recanalization was identified in 28 (83%) patients. Table 1 also compares hepatic artery thrombosis between the two groups (portal vein thrombosis versus non-portal vein thrombosis). As can be seen, no statistically significant differences were observed. However, when portal vein thrombosis was compared between the two groups, highly significant statistical differences were observed.

The characteristics of the patients with portal vein thrombosis are presented in Table 2. The most frequent extension of thrombosis was Yerdel 1 (58.8%). A portosystemic shunt was identified in 23 (68%) patients, the most frequent being splenorenal in 62% (21) of the cases, with physiological flow diameters greater than 1 cm in 26% of them. The most used type of reconstruction was resection and portoportal anastomosis in 58.8% (20/34) of patients, followed by eversion thromboembolectomy in 7 (20%) patients. There were no postoperative infections in the study.

### 3.2. Early Transplant Complications

The average hospital stay was 37 days. Eleven patients (32%) presented any early vascular complication, portal vein thrombosis being the most frequent (12%). The bivariate analysis showed a statistically significant association between portal vein thrombosis and early complications (*p* = 0.004). Considering the importance of this finding, a multivariate logistic regression analysis was performed, adjusted for the variables age, gender, weight, height, body mass index, and Yerdel. This analysis corroborated a strong association between portal vein thrombosis and early complications (OR = 3.3, 95% confidence interval 1.4–7.7; *p* = 0.006).

On the other hand, although there was a higher frequency of hepatic artery thrombosis (12%) in transplant recipients with a history of portal vein thrombosis, the difference was not statistically significant compared to the group of transplant recipients without a history of portal vein thrombosis (8%). Finally, early mortality was observed in eight patients (24%), of which two (5.9%) presented Yerdel 2.

Regarding the comparison of preoperative and postoperative portal vein thrombosis in patients with portal vein thrombosis, it was observed that only four patients presented rethrombosis.

### 3.3. Survival of Patients with Portal Vein Thrombosis

**Figure 1:**
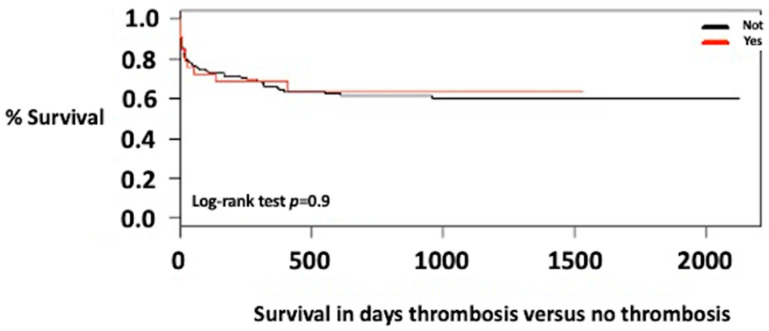
Survival of liver transplant patients with thrombosis versus no thrombosis.

The average follow-up time was 18 months (IQR = 1–29 months). The survival of patients with portal vein thrombosis was 75% at one year and 60% at 3 years; although it was slightly higher in patients without thrombosis, there were no statistically significant differences (Figure 1).

**Figure 2:**
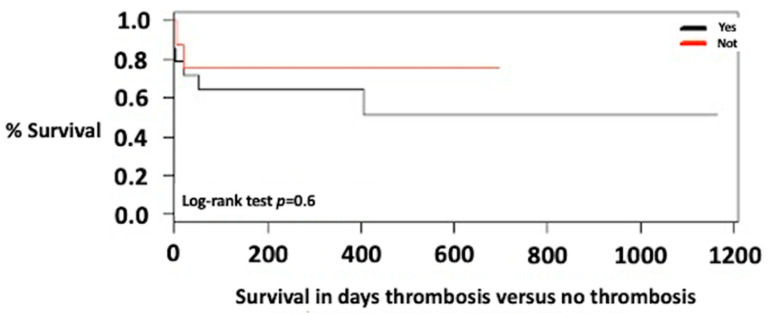
Survival of transplant patients with portal vein thrombosis with shunt versus portosystemic shunt.

In the exploratory analyses of the survival of patients with systemic or physiologic port shunts, no statistically significant differences were found (Figure 2).

**Figure 3:**
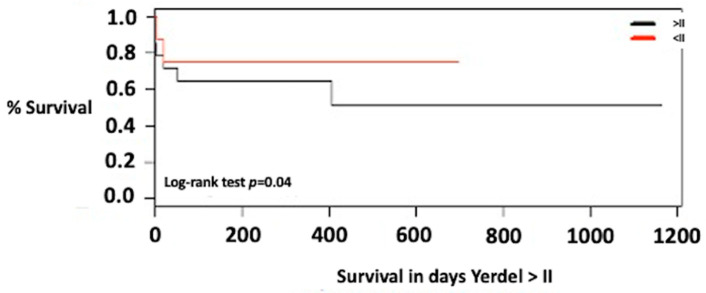
Survival of liver transplant patients with portal vein thrombosis Yerdel 1 versus Yerdel 2 or higher.

When comparing the survival of the patients according to the extension of the thrombosis, a survival of 75% at one year and at 3 years was found for Yerdel 1. For Yerdel 2 or higher, it was 65% at one year and 50% at 3 years (*p* = 0.04) (Figure 3).

## 4. Discussion

Recently, more patients with portal vein thrombosis are being accepted for liver transplantation, especially in experienced liver transplantation centers, after being considered an absolute contraindication for the procedure due to the high mortality associated with it for a long time [1,2,4]. According to early research, patients with portal vein thrombosis had lower post-liver transplant outcomes than patients without the condition. However, most studies released after 2000 have shown comparable 1-year survival in both groups, assuming an end-to-end porto-portal anastomosis can be made during liver transplantation once the thrombus has been cleared [1]. This is habitually achievable in Grade I/II Yerdel portal vein thrombosis but can be challenging in patients with diffuse (Grade III/IV Yerdel) portal vein thrombosis. Due to the poorer outcomes, the latter category of patients is still not evaluated for liver transplantation by most medical centers globally [1,3]. Our highly complex hospital is the most experienced transplant center in Colombia and one of the most relevant in Latin America. Therefore, we believe that the present study can provide knowledge on the management of portal vein thrombosis in patients with liver transplantation in this part of the world where studies in this regard are scarce.

Portal vein thrombosis in cirrhotic patients not associated with hepatocarcinoma is generated by multicausal phenomena. Among them are the increased resistance to portal flow, secondary to hepatic architectural changes (due to parenchyma extinction and aggravated by splanchnic vasodilation), additionally worsened by the loss of the delicate balance between procoagulant and antifibrinolytic elements, characteristic of the cirrhotic patient [33,34]. Its prevalence is between 2% and 26%, according to different reported series [14,15]. This information agrees with the data from the present study (17%), where the majority were identified during the pre-transplant protocol (31/34). It is noteworthy that 8.8% (3/34) had been identified at the time of the transplant, an aspect that correlates with previous results that showed an incidence of 7.4% in a 12-month follow-up period on the waiting list [35]. Given this rare but important incidental finding, the surgeon should have a pre-transplant vascular evaluation regarding the presence of the anatomy of the splenic portomesenteric axis, the presence of portosystemic shunts, and whether these are physiological or not [17]. These aspects provide tools to give an intraoperative solution to the type of portal reconstruction to be used, in case of incidental findings or progression of thrombosis.

Anticoagulation in patients with acute portal vein thrombosis is a fundamental part of their treatment, and its recommendation has a high level of evidence to achieve their recanalization [36]. However, although it is now recommended in cirrhotic patients with thrombosis on the waiting list for liver transplantation, explained by series that achieve recanalization rates ranging from 42% to 75% [15,35,37], its level of recommendation was low in the new Baveno VI consensus [38]. This information supports our finding of high recanalization (83%) in a low group of patients who received anticoagulation (28%). However, although anticoagulation is moderately useful in recanalization, it reduces the extent of thrombosis on the splenic portomesenteric axis [15,39], which may reduce the risk of complete and complex thrombosis that limits the possibility of transplantation or impact perioperative morbidity and mortality.

The presence of systemic port shunts in cirrhotic patients has a frequency between 20% and 40% [40,41]; however, its frequency or impact on complex portal vein thrombosis in patients undergoing liver transplantation has not been reported [17]. This study found a shunt frequency of 68% (23/34) in patients with portal vein thrombosis who received liver transplantation; however, the presence of a shunt did not impact the survival of liver transplant patients with portal vein thrombosis (Figure 2).

Multiple classifications have been used to assess the extent of thrombosis over the splenic portomesenteric axis. In our environment, the most widely used for standardization is that of Yerdel et al. [18], because it allows for the establishment of a more appropriate approach to the surgical treatment to follow [17]. In the present study, non-complex partial thrombosis, or Yerdel 1, was the most frequent, and this is consistent with previous studies [12,36,42]. On the other hand, the frequency of complete and complex thrombosis (Yerdel 3–4) was 0.99% (2/201), corroborating the low reported prevalence of complex thrombosis (2.2%) [14].

There were no postoperative infections in the current study. Similar results were described by Hibi et al. [43]. However, according to Yerdel et al. [18], there is a propensity for increased infectious sequelae in portal vein thrombosis patients, particularly in advanced stages of portal vein thrombosis, but the difference is not statistically significant. This is most likely because they are more fragile patients undergoing more difficult surgery with higher blood transfusion needs [18].

Although, in the present study, hepatic arterial thrombosis was 12% and 8% in the groups of patients with portal vein thrombosis and non-portal vein thrombosis, respectively (*p* > 0.05), hepatic arterial thrombosis and portal vein thrombosis are serious postoperative complications that can cause graft loss, the need for urgent retransplantation, and, in many cases, death.

Mortality at 30 days reported in our series was 24% (8/34) and varied greatly with the degree of thrombosis, being more frequent in complete and complex thrombosis; however, there were no differences in mortality with the non-thrombosis group. Different investigators reported early mortality between 13% and 30% in patients with thrombosis [16,18]. It has been reported that early mortality is lower in transplant recipients without thrombosis (7–16.5%) [14,16,18]. A previous study indicated that there are differences in early mortality in patients with portal vein thrombosis with physiologic reconstructions, compared with those without portal vein thrombosis (3.4% versus 5.1%). Furthermore, mortality was 24% (*p* < 0.001) in those with portal vein thrombosis with non-physiological reconstructions (Yerdel 3 and 4) [43]. Some benefits of the current study in clinical practice include a strong association between portal vein thrombosis and early complications. Additionally, it was observed that although the early mortality of all transplant recipients was high in the patients studied when cutting into two periods (2013–2018 versus 2019–2021), it was found that it was lower in the second period (10%). This is explained, as in the work by Yerdel et al. [18], by the variability of mortality according to the study period, a matter related to the experience of the surgical group and the volume of procedures.

The frequency of early portal vein rethrombosis occurred in 12% (4/34) of the cases in our cohort, which is consistent with the frequencies reported in different studies (between 4.7% and 36%) [18,42,43,44,45]. However, half (2/4) of the patients in the present investigation had early mortality, such as the mortality reported after portal rethrombosis, which varies between 42 and 100% [42,46]. The frequencies of rethrombosis and its associated mortality depend on the occurrence of complete and/or complex portal vein thrombosis that requires non-physiological reconstructions [46,47]. The most accepted management of rethrombosis is anticoagulation [4], with graft salvage rates reaching 46%; however, other alternatives have been reported, such as surgical revision (32%), percutaneous thrombectomy (40%), portosystemic shunts (50%), and even retransplantation [45]. In this investigation, the management offered to patients included that in which portal rethrombosis was successful in 50% of the cases, whose treatment was anticoagulation only, or anticoagulation plus percutaneous thrombectomy. Fatal outcomes occurred in those who underwent surgical revision. However, they were associated with grafts whose portal rethrombosis had a negative impact on their function, and they were associated with patients with greater systemic involvement.

The survival of patients with portal vein thrombosis after one year of follow-up was between 65% and 85%, and at 5 years between 65% and 68% [18,43,45,48]. Differences have been reported between mortality compared with transplant recipients without portal vein thrombosis. Some groups show similar survival with those without this background [49] and other series show a better survival at one year and at 5 years in those transplanted without portal vein thrombosis [18,43]. This information agrees with the findings of this study where the survival of transplant recipients with portal vein thrombosis at 1 year and 3 years was 75 and 60%, respectively. On the other hand, the explanation of the differences in survival among the transplanted patients lies in the extension of the thrombosis, and the analysis of the groups, according to the time in which they were transplanted. For example, the survival of patients with partial thromboses (Yerdel 1) was 86% at one year and 5 years, like those without portal vein thrombosis [18]. In cases of complete or complex thrombosis that required non-physiologic reconstructions, survival decreased at 1 year (50–64%), and at 5 years (47–50%) [18,43]. Another clinical benefit of the present study is that the previous findings are corroborated in our context, in which better survival was observed in transplant recipients with partial thrombosis (Yerdel 1), compared to those with total thrombosis (Yerdel 2 or higher).

While successful liver transplantation in patients with portal vein thrombosis can lead to favorable outcomes, it is important to note that the prognosis can vary among individuals. The presence of other comorbidities, the severity of liver disease, the response to immunosuppressive medications, and the occurrence of complications post-transplantation can all influence the overall prognosis. Regular follow-up and monitoring are essential to assess the long-term outcomes of patients with portal vein thrombosis after liver transplantation [1,2,3,4].

Close collaboration among hepatologists, transplant surgeons, radiologists, and other specialists is crucial in evaluating and managing patients with portal vein thrombosis undergoing liver transplantation. The decision to proceed with transplantation is based on a thorough assessment of the risks and benefits, considering the individual patient’s clinical presentation, the severity of liver disease, tumor characteristics (if present), overall health, and the specific circumstances of the portal vein thrombosis. Moreover, the expertise of the transplant center and the multidisciplinary team involved in the transplantation procedure play a significant role in the prognosis. Centers with extensive experience in managing portal vein thrombosis and performing liver transplantation have generally shown better outcomes [1].

The current study has some limitations. The first one is related to its retrospective design that prevents the establishment of a direct causal relationship. Furthermore, this is a study carried out in a single hospital center and with a small sample of patients with portal vein thrombosis, but it was obtained from more than 200 transplant patients observed over a period of 8 years.

## 5. Conclusions

In conclusion, portal vein thrombosis significantly influenced early vascular complications. Moreover, complete and complex portal vein thrombosis (Yerdels 3 and 4) have a higher early mortality, while complete portal vein thrombosis (Yerdel 2) has a negative impact on 1-year and 3-year graft survival.

## Figures and Tables

**Table 1:** Description of the general characteristics of liver transplant patients with and without a history of portal vein thrombosis.

Characteristic	No Portal Vein Thrombosis n = 167	Portal Vein Thrombosis n = 34	*p*-Value
Gender, n %			
Male	91 (55%)	22 (65%)	0.3
Female	75 (45%)	12 (35%)	0.4
Age, mean (SD)	56 (12.2)	59 (9.2)	0.1
Hospital stays, mean (SD)	24 (34.9)	37 (64.6)	0.25
Body mass index, mean (SD)	26 (4.8)	27 (5)	0.29
Hepatocellular carcinoma, n %			
Yes	59 (35%)	14 (41%)	0.65
Child			
A	36 (21%)	4 (12%)	0.28
B	73 (44%)	16 (47%)	0.86
C	58 (35%)	14 (41%)	0.64
Type of early vascular complication			
Hepatic artery thrombosis	14 (8%)	4 (12%)	0.76
Hepatic artery stenosis	1 (0.6%)	1 (3%)	0.78
Portal Vein thrombosis	2 (1%)	4 (12%)	0.006
Meld, mean (SD)	20	19	0.41
Death in the first month, n %	42 (25%)	8 (24%)	1

**Table 2:** Description of the characteristics of 34 transplant patients with portal vein thrombosis.

Characteristic	n	Percentage
Yerdel		
I	20	58.8%
II	12	35.2%
III	1	3%
IV	1	3%
Shunt presence		
Yes	23	68%
Not	11	32%
Shunt type		
Splenorenal	21	62%
Gastric	2	6%
Physiological		
>1 cm	9	26%
<1 cm	14	41%
Child		
A	4	12%
B	16	47%
C	14	41%
Early Vascular Complication		
Yes	11	32%
Not	23	68%
Type of Early Vascular Complication		
Hepatic artery thrombosis	4	12%
Hepatic artery stenosis	1	3%
Portal Vein Thrombosis	4	12%
Hepatic Vein Stenosis	1	3%
Portal vein thrombosis and Hepatic Artery	1	3%

## Data Availability

The datasets used and/or analyzed during the present study are available from the corresponding author upon reasonable request.

## References

[1] Bhangui P., Fernandes E.S., Di Benedetto F., Joo D.-J., Nadalin S. (2020). Current management of portal vein thrombosis in liver transplantation. Int. J. Surg..

[2] Agbim U., Satapathy S.K. (2020). PRO: Portal Vein Thrombosis Impacts Liver Transplantation Outcomes. Clin. Liver Dis..

[3] Teng F., Sun K.-Y., Fu Z.-R. (2020). Tailored classification of portal vein thrombosis for liver transplantation: Focus on strategies for portal vein inflow reconstruction. World J. Gastroenterol..

[4] Qi X., Dai J., Jia J., Ren W., Yang M., Li H., Fan D., Guo X. (2015). Association between Portal Vein Thrombosis and Survival of Liver Transplant Recipients: A Systematic Review and Meta-analysis of Observational Studies. J. Gastrointest. Liver Dis..

[5] Qi X., Ren W., De Stefano V., Fan D. (2014). Associations of Coagulation Factor V Leiden and Prothrombin G20210A Mutations with Budd–Chiari Syndrome and Portal Vein Thrombosis: A Systematic Review and Meta-analysis. Clin. Gastroenterol. Hepatol..

[6] Nishimura E., Misawa T., Kitamura H., Fujioka S., Akiba T., Yanaga K. (2018). A case of portal vein thrombosis caused by blunt abdominal trauma in a patient with low protein C activity. Clin. J. Gastroenterol..

[7] Dumic I., Tankosic N., Stojkovic Lalosevic M., Alempijevic T. (2017). Sport-Related Portal Vein Thrombosis: An Unusual Complication. Case Rep. Hepatol..

[8] Alzubaidi S., Patel I., Saini A., Knuttinen G., Naidu S., Kriegshuaser S., Albadawi H., Oklu R. (2019). Current concepts in portal vein thrombosis: Etiology, clinical presentation and management. Abdom. Imaging.

[9] Northup P.G., Garcia-Pagan J.C., Garcia-Tsao G., Intagliata N.M., Superina R.A., Roberts L.N. (2021). Vascular Liver Disorders, Portal Vein Thrombosis, and Procedural Bleeding in Patients with Liver Disease: 2020 Practice Guidance by the American Association for the Study of Liver Diseases. Hepatology.

[10] Fusaro L., Di Bella S., Martingano P., Crocè L.S., Giuffrè M. (2023). Pylephlebitis: A Systematic Review on Etiology, Diagnosis, and Treatment of Infective Portal Vein Thrombosis. Diagnostics.

[11] Guerra F., Dorma M.P.F., Giuliani G., Caravaglios G., Coratti A. (2023). Pylephlebitis: An uncommon complication of sigmoid diverticulitis. Am. J. Emerg. Med..

[12] Van Thiel D.H., Schade R.R., Starzl T.E., Iwatsuki S., Shaw B.W., Gavaler J.S., Dugas M. (1982). Liver transplantation in adults. Hepatology.

[13] Shaw B.W., Iwatsuki S., Bron K., Starzl T.E., Bran K. (1985). Portal vein grafts in hepatic transplantation. Surg. Gynecol. Obstet..

[14] Rodríguez-Castro K.I., Porte R.J., Nadal E., Germani G., Burra P., Senzolo M. (2012). Management of Nonneoplastic Portal Vein Thrombosis in the Setting of Liver Transplantation: A Systematic Review. Transplantation.

[15] Francoz C., Valla D., Durand F. (2012). Portal vein thrombosis, cirrhosis, and liver transplantation. J. Hepatol..

[16] Zanetto A., Rodriguez-Kastro K.-I., Germani G., Ferrarese A., Cillo U., Burra P., Senzolo M. (2018). Mortality in liver transplant recipients with portal vein thrombosis—An updated meta-analysis. Transplant. Int..

[17] Bhangui P., Lim C., Levesque E., Salloum C., Lahat E., Feray C., Azoulay D. (2019). Novel classification of non-malignant portal vein thrombosis: A guide to surgical decision-making during liver transplantation. J. Hepatol..

[18] Yerdel M.A., Gunson B., Mirza D., Karayalçin K., Olliff S., Buckels J., Pirenne J. (2020). Portal vein thrombosis in adults undergoing liver transplantation: Risk factors, screening, management, and outcome. Transplantation.

[19] Molmenti E.P., Roodhouse T.W., Molmenti H., Jaiswal K., Jung G., Marubashi S., Sanchez E.Q., Gogel B., Levy M.F., Goldstein R.M. (2002). Thrombendvenectomy for Organized Portal Vein Thrombosis at the Time of Liver Transplantation. Ann. Surg..

[20] Dumortier J., Czyglik O., Poncet G., Blanchet M.-C., Boucaud C., Henry L., Boillot O. (2002). Eversion Thrombectomy for Portal Vein Thrombosis During Liver Transplantation. Am. J. Transplant..

[21] Robles R., Fernandez J.A., Hernandez Q., Marin C., Ramirez P., Bueno F.S., Lujan J.A., Rodriguez J.M., Acosta F., Parrilla P. (2004). Eversion thromboendovenectomy in organized portal vein thrombosis during liver transplantation. Clin. Transplant..

[22] Nacif L.S., Zanini L.Y., Pinheiro R.S., Waisberg D.R., Rocha-Santos V., Andraus W., Carrilho F.J., Carneiro-D’albuquerque L. (2021). Portal vein surgical treatment on non-tumoral portal vein thrombosis in liver transplantation: Systematic Review and Meta-Analysis. Clinics.

[23] Kato T., Levi D.M., DeFaria W., Nishida S., Tzakis A.G. (2000). Liver transplantation with renoportal anastomosis after distal splenorenal shunt. Arch. Surg..

[24] Golse N., Bucur P.O., Faitot F., Bekheit M., Pittau G., Ciacio O., Antonio S.A., René A., Denis D., Didier D. (2015). Spontaneous Splenorenal Shunt in Liver Transplantation: Results of Left Renal Vein Ligation Versus Renoportal Anastomosis. Transplantation.

[25] D’Amico G., Hassan A., Diago Uso T., Hashmimoto K., Aucejo F.N., Fujiki M., Eghtesad B., Sasaki K., Lindenmeyer C.C., Miller C.M. (2019). Renoportal anastomosis in liver transplantation and its impact on patient outcomes: A systematic literature review. Transplant. Int..

[26] Lerut J.P., Lai Q., Goyet J.D.V.D. (2019). Cavoportal Hemitransposition in Liver Transplantation: Toward a More Safe and Efficient Technique. Liver Transplant..

[27] Bhangui P., Lim C., Salloum C., Andreani P., Sebbagh M., Hoti E., Azoulay D. (2017). Caval inflow to the graft for liver transplantation in patients with diffuse portal vein thrombosis: A 12-year experience. Ann. Surg..

[28] Vianna R.M., Mangus R.S., Kubal C., Fridell J.A., Beduschi T., Tector A.J. (2012). Multivisceral transplantation for diffuse portomesenteric thrombosis. Ann. Surg..

[29] Wang Z., Yang L. (2014). Gastric coronary vein to portal vein reconstruction in liver transplant: Case report. Exp. Clin. Transplant..

[30] Bhangui P., Salloum C., Lim C., Andreani P., Ariche A., Adam R., Castaing D., Kerba T., Azoulay D. (2014). Portal vein arterialization: A salvage procedure for a totally de-arterialized liver. The Paul Brousse Hospital experience. HPB.

[31] Schnickel G.T., Busuttil R.W. (2015). Portal Vein Thrombosis and Other Venous Anomalies. Transplantation of the Liver.

[32] R Core Team (2020). R: A Language and Environment for Statistical Computing.

[33] Tripodi A., Mannucci P.M. (2011). The Coagulopathy of Chronic Liver Disease. N. Engl. J. Med..

[34] Northup P.G. (2009). Hypercoagulation in Liver Disease. Clin. Liver Dis..

[35] Francoz C., Belghiti J., Vilgrain V., Sommacale D., Paradis V., Condat B., Denninger M.H., Sauvanet A., Valla D., Durand F. (2005). Splanchnic vein thrombosis in candidates for liver transplantation: Usefulness of screening and anticoagulation. Gut.

[36] de Franchis R., Faculty B.V. (2010). Revising consensus in portal hypertension: Report of the Baveno V consensus workshop on methodology of diagnosis and therapy in portal hypertension. J. Hepatol..

[37] Amitrano L., Guardascione M.A., Menchise A., Martino R., Scaglione M., Giovine S., Romano L., Balzano A. (2010). Safety and efficacy of anticoagulation therapy with low molecular weight heparin for portal vein thrombosis in patients with liver cirrhosis. J. Clin. Gastroenterol..

[38] de Franchis R., Faculty B.V. (2015). Expanding consensus in portal hypertension: Report of the Baveno VI Consensus Workshop: Stratifying risk and individualizing care for portal hypertension. J. Hepatol..

[39] Francoz C., Dondero D., Abdel-Razek W. (2008). Screening for portal vein thrombosis in candidates for liver transplantation and anticoagulation until transplantation: Results of a prospective assessment. Liver Transplant..

[40] Simón-Talero M., Roccarina D., Martínez J., Lampichler K., Baiges A., Low G., Botella E.R. (2018). Association Between Portosystemic Shunts and Increased Complications and Mortality in Patients with Cirrhosis. Gastroenterology.

[41] Gavara C.G., Bhangui P., Salloum C., Osseis M., Esposito F., Moussallem T., Lahat E., Fuentes L., Compagnon P., Ngongang N. (2017). Ligation versus no ligation of spontaneous portosystemic shunts during liver transplantation: Audit of a prospective series of 66 consecutive patients. Liver Transplant..

[42] Lendoire J., Raffin G., Cejas N., Duek F., Schelotto P.B., Trigo P., Quarin C., Garay V., Imventarza O. (2007). Liver transplantation in adult patients with portal vein thrombosis: Risk factors, management and outcome. HPB.

[43] Hibi T., Nishida S., Levi D.M., Selvaggi G., Tekin A., Fan J., Tzakis A.G. (2014). When and why portal vein thrombosis matters in liver transplantation: A critical audit of 174 cases. Ann. Surg..

[44] Ponziani F.R., Zocco M.A., Senzolo M., Pompili M., Gasbarrini A., Avolio A.W. (2014). Portal vein thrombosis and liver transplantation: Implications for waiting list period, surgical approach, early and late follow-up. Transplant. Rev..

[45] Duffy J.P., Hong J.C., Farmer D.G., Ghobrial R.M., Yersiz H., Hiatt J.R., Busuttil R.W. (2009). Vascular complications of orthotopic liver transplantation: Experience in more than 4,200 patients. J. Am. Coll. Surg..

[46] Manzanet G., Sanjuán F., Orbis P., López R., Moya A., Juan M., Mir J. (2001). Liver transplantation in patients with portal vein thrombosis. Liver Transplant..

[47] Chen H., Turon F., Hernández-Gea V., Fuster J., Garcia-Criado A., Barrufet M., Garcia-Pagán J.C. (2016). Nontumoral portal vein thrombosis in patients awaiting liver transplantation. Liver Transplant..

[48] Ravaioli M., Zanello M., Grazi G.L., Ercolani G., Cescon M., Del Gaudio M., Pinna A.D. (2011). Portal vein thrombosis and liver transplantation: Evolution during 10 years of experience at the University of Bologna. Ann. Surg..

[49] Padilla-Machaca P.M., Chaman Ortiz J.C. (2019). Portal vein thrombosis in patients undergoing to liver transplantation. Rev. Gastroenterol. Peru.

